# Genomic variant benchmark: if you cannot measure it, you cannot improve it

**DOI:** 10.1186/s13059-023-03061-1

**Published:** 2023-10-05

**Authors:** Sina Majidian, Daniel Paiva Agustinho, Chen-Shan Chin, Fritz J. Sedlazeck, Medhat Mahmoud

**Affiliations:** 1 Department of Computational Biology, University of Lausanne, 1015 Lausanne, Switzerland; 2 SIB Swiss Institute of Bioinformatics, 1015 Lausanne, Switzerland; 3 Baylor College of Medicine, Human Genome Sequencing Center, Houston, TX 77030 USA; 4 Sema4 OpCo, Inc., Stamford, CT 06405 USA; 5 Department of Computer Science, Rice University, 6100 Main Street, Houston, TX 77005 USA; 6 Department of Molecular and Human Genetics, Baylor College of Medicine, Houston, TX USA

**Keywords:** Genetic variation, SNPs, Indels, Structural variant, Benchmark datasets, Medical genes, Sequencing technology

## Abstract

Genomic benchmark datasets are essential to driving the field of genomics and bioinformatics. They provide a snapshot of the performances of sequencing technologies and analytical methods and highlight future challenges. However, they depend on sequencing technology, reference genome, and available benchmarking methods. Thus, creating a genomic benchmark dataset is laborious and highly challenging, often involving multiple sequencing technologies, different variant calling tools, and laborious manual curation. In this review, we discuss the available benchmark datasets and their utility. Additionally, we focus on the most recent benchmark of genes with medical relevance and challenging genomic complexity.

**Supplementary Information:**

The online version contains supplementary material available at 10.1186/s13059-023-03061-1.

## Background

Novel bioinformatics methods and DNA sequencing technologies are being developed regularly. This enables more accurate detection of genetic variations with higher resolution [1–3]. Nevertheless, as Baron Kelvin proclaimed, “If you cannot measure it, you cannot improve it,” and therefore, it is crucial to assess the accuracy of identifying variants, paving the way to advance the field of genomics in sensitive clinical applications [4, 5]. Correct measurement is at the mercy of having appropriate controls, such as benchmark datasets, which are the yardstick to evaluate bioinformatics methods and the performance of sequencing technologies [4]. In other words, benchmark datasets containing well-established variant calls are needed to develop, optimize, and analytically validate variant detection methods, which can ultimately be translated into systematic research studies and clinical practices**.** Such benchmark datasets are useful for a diverse range of researchers involved in sequencer manufacturing, library preparation, bioinformatics method development, and clinical studies.

Historically, multiple different benchmark datasets have been created using simulated data pipelines [6–9]. While these simulated datasets typically suffer from a simplistic representation of real genomics data, they often prove to be useful, in metagenomics [10] for example. Synthetic benchmark datasets [11] (e.g., spike in [12] or a specific artificial sample) overcome this simplicity issue partially, but often still suffer from a less reliable representation of the underlying challenges found in real datasets. More recently, benchmark datasets created with fully characterized “real” data have emerged. These rely on stable cell lines obtained by genetic manipulation of primary cells from an individual and the usage of multiple sequencing technologies [11, 13, 14]. The benefits from using real datasets are highly dependent on how accurately it was characterized. This characterization is the most challenging part of creating such a benchmark dataset, but it best represents the challenges that scientists face when analyzing their genomic samples [15].

Here, we describe the current state-of-the-art genomic benchmark datasets that are publically available to the community. We give special attention to the most recent Genome in a Bottle (GIAB) benchmark, focusing on 386 Challenging Medically Relevant Genes (CMRG) [16]. This represents the currently most challenging benchmark, including 17,000 single-nucleotide variants (SNVs), 3600 small insertions and deletions (indels, 1–49 bp long), and 200 structural variants (SVs 50 + bp) across 273 genes, most of which are in highly repetitive or complex regions. Furthermore, we discuss the impact and emergence of new sequencing technologies, as well as challenges and opportunities for future genomic benchmark datasets. This includes recent improvements in new sequencing technology and updates on reference genomes and their impact on creating such datasets. This review ends with an overview of tools for benchmark curation and their challenges, followed by the discussion of potential future directions for benchmarks.

## Overview of genomic benchmarks

Nowadays, we have multiple benchmark datasets available for the assessment of genomic variations (Table 1). A genome variant benchmark dataset serves as a vital resource for evaluating the effectiveness and accuracy of newly developed variant callers, whether they focus on small or large genomic alterations. This dataset comprises known curated genomic variants. To ensure a comprehensive assessment, it is crucial for the benchmark dataset to include relevant information about the specific genomic regions associated with these variants. This distinction is essential as it helps differentiate these regions from those that were not considered in the benchmark dataset, such as regions that are not assembled or lack support from multiple callers [16]. This information is typically captured and represented in a BED file format, ensuring that researchers have precise details regarding the genomic coordinates and regions associated with each variant.

**Table 1:** Chronological order of benchmark datasets for different variant types including point mutation, insertion, deletions, and structural variant for healthy and patient samples

Publication Title	Project name	Year	Doi	PMID	Data	Number of samples	Technology	Status Sample	Cell	Variants	Reference included %	Reference
A comprehensive catalogue of somatic mutations from a human cancer genome	The catalogue of somatic mutations	2010	https://doi.org/10.1038/nature08658	20016485	Whole genome sequencing	1 sample (COLO-829)	Illumina GAII	Patient	Somatic	SNV and indel < 50 bp	N/A	NCBI36
A map of human genome variation from population-scale sequencing	1000 Genomes Project	2010	https://doi.org/10.1038/nature09534	20981092	Whole genome sequencing, exon-targeted sequencing	882 samples (low-coverage whole-genome sequencing of 179 individuals; high-coverage sequencing of two mother–father–child trios; exon-targeted sequencing of 697 individuals)	454 GS FLX, Illumina Genome Analyzer, and AB SOLiD System	Healthy	Germline	SNV and indel < 50 bp	85	NCBI36
Integrating human sequence data sets provides a resource of benchmark SNP and indel genotype calls	GIAB v.2.19	2014	https://doi.org/10.1038/nbt.2835	24531798	Whole genome sequencing, exome sequencing	1 sample (NA12878, 11 whole-genome and 3 exome)	454, Complete Genomics, Illumina, Ion Torrent and SOLiD 4	Healthy	Germline	SNV and indel < 50 bp	77	GRCh37
svclassify: a method to establish benchmark structural variant calls	svclassify	2016	https://doi.org/10.1186/s12864-016-2366-2	26772178	whole genome sequencing	1 sample (NA12878)	Illumina HiSeq, Moleculo and PacBio	Healthy	Germline	SV and indel < 50 bp	N/A	GRCh37
Extensive sequencing of seven human genomes to characterize benchmark reference materials	GIAB Public Data	2016	https://doi.org/10.1038/sdata.2016.25	27271295	Whole genome sequencing	7 samples (HG001-7)	10xGenomics, BioNano, Complete Genomics (paired-end and LFR), GemCode WGS, Illumina (exome and WGS paired-end, mate-pair, and synthetic long reads), Ion Proton exome, ONT, PacBio, and SOLiD	Healthy	Germline	SNV, indel, and SV	N/A	GRCh37
A reference data set of 5.4 million phased human variants validated by genetic inheritance from sequencing a three-generation 17-member pedigree	Platinum Genomes	2017	http://dx.doi.org/10.1101/gr.210500.116	27903644	Whole genome sequencing	2 samples (2 individuals with benchmarks, but using short-read WGS from 11 children and 4 grandparents from CEPH pedigree 1463)	Illumina	Healthy	Germline	SNV and Indel < 50 bp	96.7	GRCh37
A synthetic-diploid benchmark for accurate variant calling evaluation	CHM-eval, aka Syndip	2018	https://doi.org/10.1038/s41592-018-0054-7	30013044	Whole genome sequencing	2 samples (Synthetic mixture of two effectively haploid hydatidiform mole cell lines)	PacBio CLR	Haploid cell lines	Germline	SNV, indel > 1 bp, and SV	96	GRCh37 and GRCh38
An open resource for accurately benchmarking small variant and reference calls	GIAB v.3.3.2	2019	https://doi.org/10.1038/s41587-019-0074-6	30936564	Whole genome sequencing	7 samples (HG001-7)	10 × Genomics, Illumina, Complete Genomics, Ion Torrent and SOLiD 4	Healthy	Germline	SNV and indel < 50 bp	85.4	GRCh37 and GRCh38
A robust benchmark for detection of germline large deletions and insertions	NIST v0.6 SV benchmark set	2020	https://doi.org/10.1038/s41587-020-0538-8	32541955	Whole genome sequencing	1 sample (HG002)	10 × Genomics, Illumina, PacBio CLR, ONT	Healthy	Germline	indel > = 50 bp	86	GRCh37
A diploid assembly-based benchmark for variants in the major histocompatibility complex	MHC benchmark	2020	https://doi.org/10.1038/s41467-020-18564-9	32963235	Whole genome sequencing	1 sample (HG002)	10 × Genomics, PacBio HiFi, and ONT	Healthy	Germline	SNV and indel < 50 bp	N/A	GRCh37 and GRCh38
Establishing community reference samples, data and call sets for benchmarking cancer mutation detection using whole-genome sequencing	SEQC2 Tumor-normal	2021	https://doi.org/10.1038/s41587-021-00993-6	34504347	Whole genome sequencing, exome sequencing	1 tumor/normal cell line pair	10 × Genomics, Illumina, Ion Torrent, and PacBio HiFi	Patient	Somatic	SNV and indel < 50 bp	N/A	GRCh38
A verified genomic reference sample for assessing performance of cancer panels detecting small variants of low allele frequency	SEQC2 Cancer panel	2021	https://doi.org/10.1186/s13059-021-02316-z	33863366	Targeted sequencing	Mixed tumor cell lines	Targeted Illumina Sequencing	Patient	Somatic	SNV and indel	N/A	GRCh37 and GRCh38
Benchmarking challenging small variants with linked and long reads	GIAB v.4.2.1	2022	https://doi.org/10.1016/j.xgen.2022.100128	36452119	Whole genome sequencing	7 samples (HG001-7)	10 × Genomics, Complete Genomics, Illumina, PacBio HiFi	Healthy	Germline	SNV and indel < 50 bp	92.2	GRCh37 and GRCh38
Curated variation benchmarks for challenging medically relevant autosomal genes	CMRG v1.00	2022	https://doi.org/10.1038/s41587-021-01158-1	35132260	Whole genome sequencing	1 sample (HG002)	PacBio HiFi	Healthy	Germline	SNV and SV	N/A	GRCh37 and GRCh38
A multi-platform reference for somatic structural variation detection	Somatic SV truth set	2022	https://doi.org/10.1016/j.xgen.2022.100139	36778136	Whole genome sequencing	1 sample (COLO-829)	10xGenomics, Bionano, Illumina, ONT, PacBio	Patient	Somatic	SV and indel	N/A	GRCh37 and GRCh38
Haplotype-resolved assemblies and variant benchmark of a Chinese Quartet	Chinese Quartet	2022	https://doi.org/10.1101/2022.09.08.504083	N/A	Whole genome sequencing	Two monozygotic twin daughters and their biological parents	Illumina, BGI, PacBio, and Oxford Nanopore Technology	Healthy	Germline	SNVs, indels, and SVs	N/A	GRCh38

These genomic variations can be categorized as SNVs, indels, and SVs [17, 18]. Methodologies used to identify these different variations vary [1, 17]. Their individual benchmark datasets are typically kept separated along with their unique benchmarking tools.

Although attaining 100% accuracy and sensitivity is challenging due to technological limitations, benchmark datasets strive to provide the highest accurate representation for genomic variations. To achieve this, creating a benchmark dataset involves utilizing diverse sequencing technologies such as long-reads, short-reads, and linked-reads, each with varying insert sizes and high coverage. Additionally, a range of variant calling tools and methods that rely on both mapping and assembly techniques are integrated to mitigate methodological biases. The key ingredient, however, is the manual and often wet lab assessment of the variance across a community effort [14].

In the construction of a benchmark dataset, several other criteria are carefully considered. For instance, regions where callers exhibit systematic errors or misrepresent genotypes are typically excluded [11]. Similarly, areas demonstrating high complexity may also be excluded to ensure a more accurate representation of genomic variations [14]. These selection criteria help to enhance the quality and reliability of the benchmark dataset, providing researchers with a robust and comprehensive resource for evaluating variant calling algorithms.

**Fig. 1:**
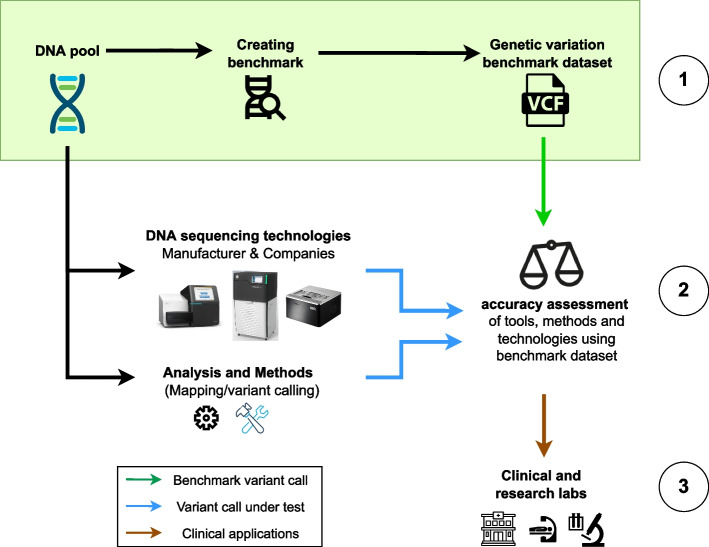
Importance of benchmark datasets in genomics and clinical setting. These datasets are beneficial in assessing different tools and methods. First, a benchmark dataset is established by using a myriad of dependable variant calling methods, forming a collection of reliable variant calls, normally stored as a VCF file (green box; step 1). New DNA sequencing technologies could be evaluated by comparing their corresponding called variants with the benchmark dataset on the same DNA sample. This showcases the reliability of their sequenced reads. The same process can be used to test new algorithms developed for read alignment or variant calling (step 2). Clinical research studies also benefit from such benchmark datasets, by incorporating newly well-established variant calling methods and sequencing technologies (step 3)

The common practice when trying to evaluate new variant calling methods is to order a DNA sample (e.g., the HG002 sample), from institutions such as the GIAB Consortium of the National Institute of Standards and Technology (NIST) or the Coriell Institute, sequence it and identify variants (Fig. 1). These samples are stored and can be obtained either as dried DNA or as immortalized cell lines. These cell lines were obtained by using the Epstein-Barr virus to perform genetic manipulation on either B lymphoblastoid or induced pluripotent stem cells from individuals [19]. When it comes to choosing the sequencing approach, any technology can be used, from short reads (e.g., Illumina) to long read (e.g., ONT: Oxford Nanopore Technologies or PacBio: Pacific Biosciences), or even optical mapping (Bionano) or other technologies [20, 21].

After sequencing, essential analysis steps including read alignment or genome assembly should be performed together with variant calling [22–24] to obtain a result that can then be compared against the benchmark dataset (e.g., the GIAB benchmark of SNVs or SV for the HG002 sample, see Fig. 1). This is done over specific SNVs or SVs benchmark methods that compare the results with that of the benchmark and provide a clear metric on the calls (precision and recall/sensitivity) of the analysis. This provides a comprehensive insight if the sequencing technology and the computational pipeline being tested are reliable. Depending on the sequencing method of choice, some parameters need to be adjusted during the evaluation of the data, for example, the minimum size of the variants for optical mapping or the constraints of sequenced resolved representation. Over the past years, this process not only helped establish pipelines and technologies, but also drove the development of new sequencing approaches [15].

The curation of such benchmark datasets is often a tedious process and typically involves multiple sequencing technologies to leverage their strengths, overcome each other’s limitations, and avoid any systematic errors from a single platform. Short-read sequencing (e.g., Illumina) is still one of the most accurate technologies and is well-established to identify SNVs and especially indels [25, 26]. However, short-read methods often struggle to characterize repetitive regions of the genome and have become known to also have limitations for SV detection [1].

**Fig. 2:**
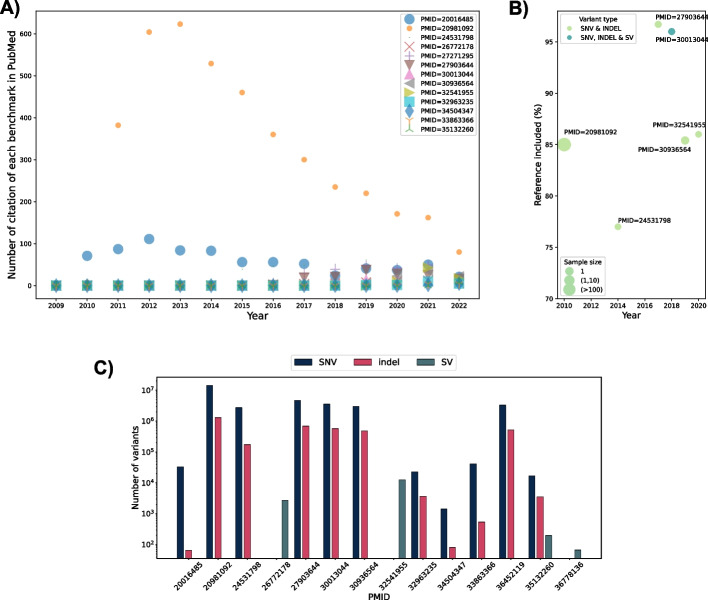
**A** Number of citations for benchmark studies using Entrez submodule of the BioPython package [31]. **B** The percentage of reference genome that is included in different benchmark studies is shown for different benchmark datasets. Besides, the sample sizes are also depicted as point size, which vary for different datasets. Another difference across them is the variant types that are included. Some benchmark datasets characterize SNVs and indels, while others cover SVs as well. **C** The number of variants (SNVs, SVs, and indels) for each benchmark dataset is represented on the *y*-axis, while the publication PMID is displayed on the *x*-axis

Since genome sequence began to be an informative resource for clinical diagnosis [27, 28], there have been several endeavors either to produce a benchmark dataset or to identify regions of complexity to reduce false-positive variants (Table 1; Fig. 2). Many of these benchmark papers have been highly cited (Fig. 2A) but differ significantly in the ratio of the reference genome they cover (Fig. 2B). Table 1 holds detailed information across the available benchmarks. Historically, Zook et al. suggested the first SNVs and indels benchmark dataset for the sample NA12878/HG001 [13]. They integrated five sequencing technologies across 14 datasets, different aligners, and variant callers. In addition, they made the data publicly available [13]. In 2016, Mandelker et al. introduced an exome-wide catalog representing high homologous exome regions to laboratories using short reads to identify variants correctly, especially for diagnostic applications [29]. There have been different attempts to enhance or produce benchmark datasets including the Genome in a Bottle Consortium (GIAB) using alignment, and de novo assembly [13, 19] and Platinum Genome [30], which are limited to specific regions. However, the Platinum Genome benchmark dataset introduced biases towards easily accessible genomic regions by only considering consensus variants from all algorithms. Thus, others suggested using the hydatidiform mole Chm13 to provide a variant benchmark, which only represents homozygous SNVs. This leads to mitigating short variant caller biases based on de novo PacBio assembly methods [11].

Later, Zook et al. enhanced the GIAB benchmark datasets by using linked-read sequencing in addition to short-read and enlarging the benchmarked regions by 12% [4]. They used two technologies in the latest version of GIAB (v.4.2.1) for sequencing seven samples (HG001, HG002, HG003, HG004, HG005, HG006, and HG007) with linked [32] and long-read [33] to characterize segmental duplication and hard-to-align regions which were traditionally often avoided. Using the previously mentioned technologies, the authors were able to add 16% more exonic regions, many of which are medically important [14]. In total, they have added more than 300,000 SNVs and 50,000 indels that were not available in the previous version; v.3.3.2 [4]. Additionally, they established a benchmark across the Major Histocompatibility Complex (MHC) region [34]. It is of note that each benchmark dataset covers a portion of the reference genome, which varies from 77 to 96% (Fig. 2B). It is important to note that benchmarks can vary significantly in terms of the number of variants they include, ranging from a few tens to millions [14, 15] (Fig. 2C). These benchmarks have been of great interest to the community as being cited by thousands of other studies (Fig. 2A). While in this review, we focus on genomic benchmarks, it is important to note that there exist additional benchmark datasets that play a pivotal role in evaluating RNA-sequencing tools. Specifically, Tang et al. have provided a benchmark dataset and a tool for evaluating the performance of RNA-seq quantification. They presented statistical summaries in terms of specificity and sensitivity at both the transcript level and gene level [35]. Another notable benchmark dataset focuses on simulating RNA-seq count data by considering two distributions: negative binomial and log-normal. This dataset was employed to compare the performance of various tools in identifying differentially expressed genes [36]. Moreover, to evaluate the effectiveness of between-sample normalization methods, an integral step in RNA-seq data analysis, an experimental ground truth was established by compiling publicly available RNA-seq assays with external spike-ins [37]. These spike-ins, typically added to biological samples at known concentrations, provide a reliable reference for evaluation. Finally, RNA counting in single-cell RNA-sequencing (scRNA-seq) also benefited from spikes [38]. However, there has been a controversy about the usefulness of this approach; see [12, 39, 40] for further discussion.

## Benchmarks for challenging medically relevant genes

**Fig. 3:**
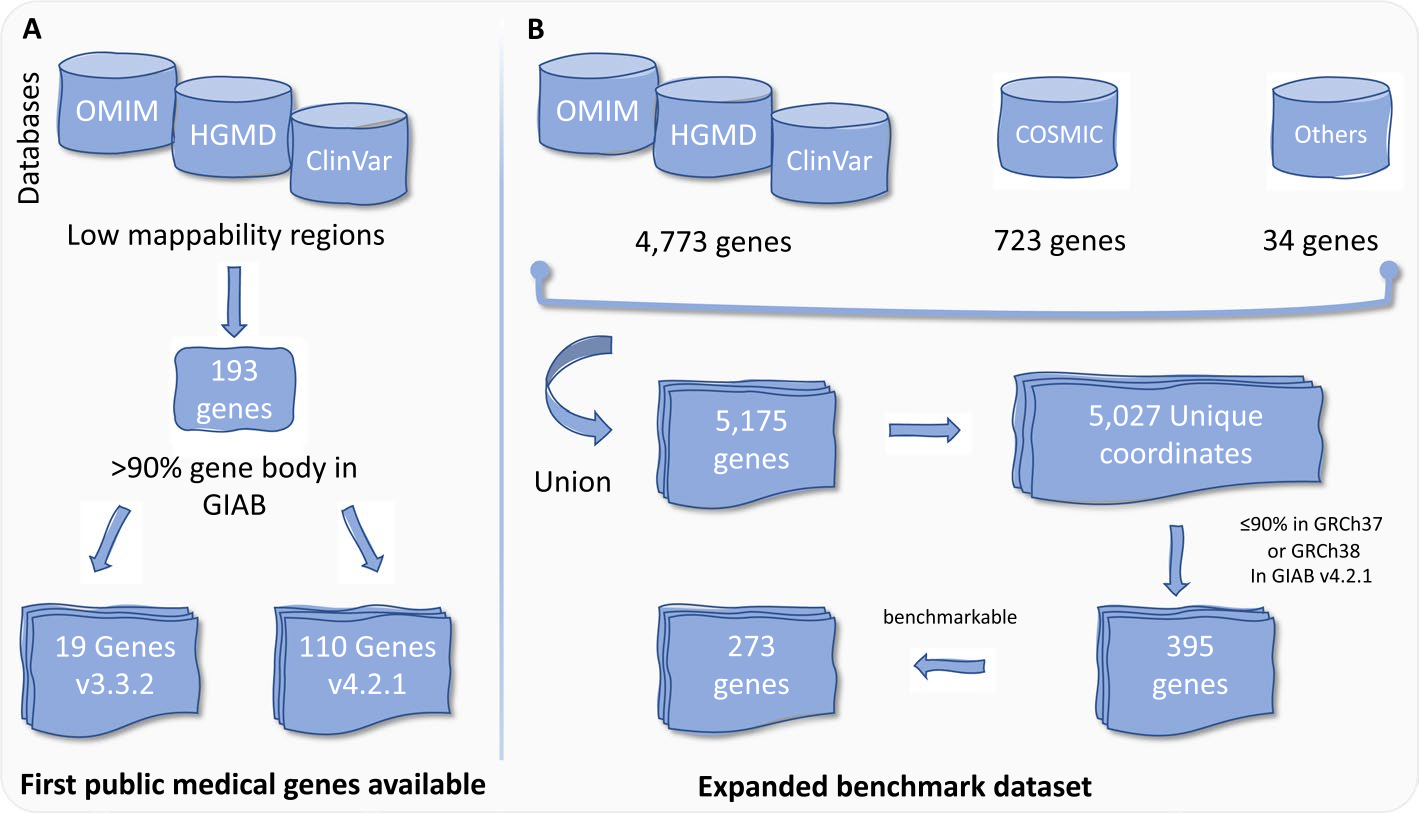
**A** Mandelker et al. [29] selected 193 challenging medical genes from three public databases that are concerned with genes causing diseases (OMIM, HGMD, and ClinVar) based on their low mappability and the percentage of gene body and number available in other GIAB dataset. **B** The development of the 273 genes dataset

Several initiatives and consortia, including GIAB, have set ambitious objectives to develop benchmark datasets of SNVs, indels, and SVs [13, 17–19, 34, 41] (Table 1). These efforts play a substantial role in the development of tools and medical research pipelines, in addition to quality control analysis. Mandelker et al. introduced one of the first lists of medically relevant genes that were difficult or impossible to analyze via the sequencing technology of their time [29]. This list includes 193 genes originating from Online Mendelian Inheritance in Man (OMIM), Human Gene Mutation Database (HGMD), and ClinVar databases (Fig. 3A). The challenge was that short reads could not resolve these genes due to their complexity (e.g., repetitive regions). Thus, they were identified as medically relevant genes with low mappability (Fig. 3A). Around 88% of these genes were then included in the GIAB benchmark database version 4.2.1 thanks to the use of linked- and long-read technologies [14]. Using HiFi long-read [32], Wagner et al. assembled a list of 5175 genes from COSMIC, OMIM, HGMD, and ClinVar databases, adding to those genes commonly tested in inherited diseases [16] (Fig. 3B). Only 5027 (4697 autosomal genes) have coordinates on GRCh38 genome assembly, and they ended up with 395 genes that have at maximum 90% of the gene body included in either GRCh37 or GRCh38 (386 if evaluating GRCh38 alone). They managed to resolve these 395 genes by the genome assembly approach using the Hifiasm assembler [42]. Of note, the above-mentioned genes are hard to analyze because of their complexity rather than consider the relative medical importance of the original 5175 genes. Finally, they managed to characterize 273 genes from the haplotype-resolved whole-genome assembly. Moreover, they reported more than 17,000 SNVs and 3600 indels plus 200 SVs for GRCh37 and GRCh38 [16].

The Challenging Medically Relevant Genes (CMRGs) is a benchmark dataset created specifically for the purpose of testing new tools [16]. It is a list of medically relevant genes of high complexity. For a gene to be included, the presence of its whole sequence plus 20 kb flanking regions on both sides is required in a single assembled contig. In addition, it needs to be aligned as one contig to GRCh37 and GRCh38 with no breaks. Albeit it may overlap with segmental duplications. Only 273 out of 395 genes described in the CMRG study by Wagner et al. were fully resolved and included in the CMRG benchmark dataset [16]. It is worth mentioning that at least 15% of the gene body of 99% of the 273 genes are either challenging to sequence or contain challenging variants to detect (due to the low mappability of these regions and the presence of repeats). In addition, 11% of CMRG indels are with a size of > 15 bp, making it challenging for tools to detect them and lowering their precision and recall. The other 122 genes of the list of 395 genes are absent from the benchmark set for various reasons. One reason is the shortcomings of the reference genomes, which include gaps in the reference sequence, being resolved only on one of the references but not the other (as happened for the *KCNJ18* gene), or duplications in HG002 compared to GRCh38 [16]. Another reason is having multiple contigs or suffering from multiple possible forms of representations (e.g., *LPA* and *CR1)*, hampering a correct benchmarking [43]. The remaining 273 CMRGs represent hard-to-assess regions of the genome that are important to obtain correct variant information and are thus challenging current available methodologies from sequencing, alignment up to variant calling and representation.

## Towards clinical usability and implementation of variation benchmarks

**Fig. 4:**
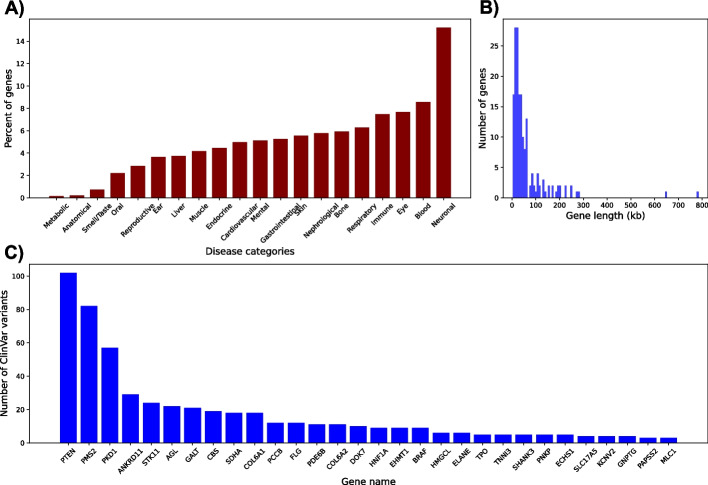
**A** Percentage of genes from Challenging Medically Relevant Genes (CMRG) list per disease category. **B** Histogram of gene length of CMRG list. **C** Number of ClinVar variants per gene for the top 30 genes in the CMRG list. Note that these variants are not part of the benchmark

In contrast to previous genome-wide efforts, the CMRGs benchmark dataset (Fig. 4) is not focused on resolving the largest portion of the genome, but challenging genes with medical importance that were not fully resolved (Fig. 3A) in previous benchmark efforts from GIAB [16]. These genes vary in sizes and complexity (Fig. 4B) and thus represent unique challenges for the analysis and sequencing technologies. Each gene in the list of CMRGs has been studied in-depth in the literature and is related to one or multiple diseases. This is also exemplified by the number of ClinVar (i.e., a database to include variations and their impact on diseases) variants that are overlapping these genes (Fig. 4C). Out of 386 total resolved genes found in GRCh38 alone, 208 of them are correlated with neuronal diseases, such as KBG syndrome (associated with the genes *ANKRD11* and *CDH15* from CMRGs), neurofibromatosis (*APOBEC1*), filamentary keratitis (FLG), and spinal muscular atrophy (*SMN1*), based on the GeneCards database [44] (Fig. 4A). Particularly, the variations of *SMN1* were investigated thoroughly in the CMRG study, as this gene has been of keen interest in the community. *SMN1* resides within a large segmental duplication on chromosome 5 making it a challenging case for variant calling. It is known that the biallelic pathogenic variants in *SMN1* can result in spinal muscular atrophy [45]. This progressive disorder is identified by muscle weakness and atrophy because of neuronal cell loss in the spinal cord [46–48]. Additionally, 117 of the genes in the CMRGs list are correlated with blood, eye, and immune diseases. Genes related to respiratory, skeletal, nephrological, and skin diseases are also included in this list.

Cardiovascular disease is another category also covered in the CMRG gene list. That includes atypical coarctation of aorta (*RNF213*), right bundle branch block (*TRPM4*), and pseudo-von Willebrand disease (*GP1BA*), as described in the GeneCards database. The third category that we mention here is immune diseases. Autoimmune lymphoproliferative syndrome (*CASP10*, *CD4*), neutropenia (*G6PC3*, *ANKRD11*, *TYMS*), anemia, autoimmune hemolytic (*CD4*, *RHCE*), and chronic granulomatous disease (*NCF1*) were found related to this category in the literature. Importantly, *NCF1* is known to be associated with 20% of cases of chronic granulomatous disease. Finally, several genes in the CMRG list had been surveyed in cancer-related studies, including colon adenocarcinoma (BAX), colorectal cancer (*BAX*, *BRAF*), hepatocellular carcinoma (*AXIN1*, *TERT*), adenocarcinoma (*BRAF*, *H19*), and prostate cancer (*BAX*, *PTEN*). Phosphatase and tensin homolog (*PTEN*), a tumor suppressor gene, is mutated in many cancers [49]. Specifically, it is commonly inactivated or lost in breast and ovarian cancers [50]. This gene may be a target for tandem duplications generating out-of-frame exon duplications [51].

Importantly, several hundreds of ClinVar variants intersect with the CMRGs list, as depicted in Fig. 4C. All in all, the CMRGs list with their genetic variations is a precious resource paving the way for answering a diverse range of clinical research questions.

## Emerging sequencing technologies

There is a cyclic reliance between the development of benchmark datasets and the emergence of new sequencing technologies and bioinformatic methods to detect variants. While in former times, the methods and technologies were developed without benchmarks, this has dramatically changed these days as benchmarks provide a form of presenting emerging technologies. In turn, once these new technologies are established, they are often integrated to form novel benchmarks. In this section, we describe novel approaches and technologies in DNA sequencing.

While short-read technologies such as Illumina’s exome sequencing are widely used for studying many genetic diseases because of their low cost and high accuracy, they still have their limitations [1, 52, 53]. Other sequencing technologies such as long-read sequencing technology have the potential to delineate a large number of SVs that could be contributing to some diseases and were undetected with short-read sequencing [54]. The two main companies that manufacture long-read DNA sequencing devices are PacBio and ONT. Using the single molecule real-time (SMRT) technology, the PacBio system produces high-fidelity (HiFi) reads which are around 15 kbp and with an error rate of less than 1% [33]. On the other hand, the ONT PromethION platform typically produces ~ 20kbp reads [55] and could reach up to 4 million base pair (Mbp) [56] with higher throughput at a lower cost, but they are less accurate (5% error rate) than HiFi reads [52, 55]. Nevertheless, both platforms perform similarly when it comes to identifying SVs [52, 57].

The ONT MinION instrument is a portable and economical sequencer. It has a DNA yield of around 20 gigabases providing a ~ 7 × coverage of the human genome [58, 59], which is insufficient for variant calling in poorly covered regions [60]. Nevertheless, it can also perform targeted enrichment during sequencing, which is possible thanks to the adaptive sampling technology [61]. It allows the pore to selectively sequence DNA molecules based on genomic regions of interest [61]. As the DNA molecule is sequenced, the nascent sequencing read is analyzed, and the software can opt to reject it, forcing the nanopore to eject the DNA molecule before sequencing is finished, opening space for sequencing a different DNA molecule. There are two recent approaches for adaptive sequencing: UNCALLED [58] and Readfish [58, 59]. Thus, adaptive sequencing can reduce the costs and lab work to sequence genes of interest.

In October 2022, PacBio launched a new sequencing instrument for long reads called Revio. This is an upgrade in capacity having four SMRT cells run in parallel, with each having a capacity of 25 million zero-mode waveguides (previously 8 million). Thus, the Revio extends the yield over the Sequel IIe by around 15 times, enabling a reported maximum of 1300 human whole genomes sequenced per year at 30 × coverage [62].

Long-read sequencing, regardless of whether it is PacBio or ONT, has played a pivotal role in addressing key challenges in genomics. It has been instrumental in filling gaps within the human genome, allowing us to obtain a comprehensive understanding of its structure for the first time in history.

Moreover, as we previously highlighted, the utility of long-read sequencing extends to the precise delineation of SVs, as well as the identification of SNVs and indels within the complex human genome regions (repetitive or duplicated regions). The application of long-read sequencing technology has had far-reaching implications, particularly in the field of medicine. It has played a crucial role in resolving numerous medical cases by facilitating the identification of disease-causing genetic variations, allowing for accurate diagnosis, such as solving CGG-repeat expansion in the fragile X gene [17, 48, 57–62]. Furthermore, new sequencing technologies emerged that also utilize genomic benchmark datasets to showcase their advantages [15, 63]. These technologies will likely be necessary to further improve current benchmark datasets. Here, we describe five novel sequencing technologies: AVITI, Illumina Complete Long-Read technology, TeLL-seq, SBB, and Ultima (Table 2). These technologies provide sequencing reads at a lower cost or higher quality, paving the way for better service in both research and clinical applications. Nevertheless, independent benchmarking is currently often sparse, so we rely here often on reports from different companies.

**Table 2:** Comparing different whole genome sequencing technologies

Technology	Company	Device name	Read length (bp)	Input DNA (microgram)	Cost	Sequencing time (hour)
Sequencing by synthesis	Illumina	NovaSeq 6000	100–300	0.3^e^	$	44^d^
Single Molecule Real-Time	PacBio	Sequel IIE	~ 15 k	5^b^	$$$	30^c^
Nanopore	ONT	PromethION	Up to 4 m	30^f^	$$	60
Tell-seq	Universal Sequencing Technology	TELL-Seq Library Prep Kit	100 k range information	5ng^g^	$^i^ + Illumina cost	3 + Illumina run
Illumina Complete Long-Read technology (previously announced as “Infinity”)	Illumina	N/A	Up to 10 k	50ng^j^	$	N/A
AVITI	Element	Element AVITI System	2*150 or 10 k	0.1–0.5	$	48^a^
Ultima	Ultima Genomics	UG 100	300	0.25	$^h^	20
Sequencing by binding	PacBio (formerly Omniome)	N/A	200	N/A	N/A	N/A

^a^https://miroculus.com/wp-content/uploads/2022/07/Elemet-AVITI-App-Note-03A.pdf

^b^https://www.pacb.com/wp-content/uploads/Procedure-Checklist-Preparing-HiFi-SMRTbell-Libraries-using-SMRTbell-Express-Template-Prep-Kit-2.0.pdf

^c^https://www.pacb.com/technology/hifi-sequencing/sequel-system/

^d^https://emea.illumina.com/systems/sequencing-platforms/novaseq.html

^e^https://www.illumina.com/content/dam/illumina/gcs/assembled-assets/marketing-literature/illumina-dna-pcr-free-loading-concentration-tech-note-770-2020-007/illumina-dna-pcr-free-loading-concentration-tech-note-770-2020-007.pdf

^f^https://nanoporetech.com/sites/default/files/s3/literature/PromethION-brochure.pdf

^g^https://sagescience.com/wp-content/uploads/2020/03/TELL-Seq_AGBT_2020_print.pdf

^h^https://www.science.org/content/article/100-genome-new-dna-sequencers-could-be-game-changer-biology-medicine

^i^900$ for the kit https://www.universalsequencing.com/shop

^j^https://www.illumina.com/products/by-brand/complete-long-reads-portfolio.html

AVITI is a sequencing system commercialized by Element Biosciences based on Avidity Chemistry [64]. In this technology, DNA attached to the flow cell is identified by a multi-pronged scaffold carrying fluorescence which is then measured by an optical imaging system, which needs fewer chemical reagents. This reduces the cost and simultaneously provides flexible read length and highly accurate sequencing data. The sequencing reads could range from 300 to 10 kbp, with a maximum throughput of 800 million reads for each flow cell [65]. AVITI sequencing offers the advantages of accurate and cost-effective short-read representation, along with a low read duplication rate. These characteristics make AVITI likely playing a role in the detection of mosaicism in metagenomics studies.

Illumina Complete Long-Read technology (previously announced as “Infinity”) is a novel long-read sequencing assay that is being developed by Illumina using the sequencing by synthesis (SBS) chemistry. Of note is that these Complete Long-Read (CLR) should not be confused with PacBio Continuous Long-Read (CLR) technology [1]. The sequencing reads of Infinity can be up to 10 kbp long, requiring 90% less DNA input compared to other long-read technologies. The company claimed that the throughput is ~ 10 × higher than that of traditional long-read technologies. Likewise, it can be used on Illumina NovaSeq 6000 sequencing system machines [66, 67]. This technology will play a crucial role in the assessment of repetitive and highly complex regions, all while maintaining cost-efficiency and requiring a small amount of input DNA.

A recent technology called transposase enzyme-linked long-read sequencing (TELL-seq) commercialized by the Universal Sequencing Technology (UST) company enables barcoding of as little as nanograms of genomic DNA in a single PCR tube with 3 h library construction, without any dedicated specialized instrument. This can generate over 100 kb of long-range sequencing information via linked reads. In the PCR tube, millions of clonally barcoded beads are used to uniquely barcode long DNA molecules in an open bulk reaction without dilution and compartmentation [68]. The de novo assembly of the human genome, structural variant detection, short tandem repeat detection, and MHC phasing of the NA12878 sample are the major analyses performed using this technology [68, 69]. It provides a cost-effective alternative to native long-read sequencing methods such as (PacBio and ONT).

Although new long-read technologies are being developed, new short-read systems are also under their way. Sequencing by binding (SBB) is a new method of short-range sequencing which was first proposed by Omniome company and was acquired by PacBio in 2021. The technology is based on the fact that binding of bases on the gold sensing surface triggers strong spectral variations within the nanohole optical response [58, 61, 70]. The analysis provided by PacBio shows a better variant calling performance in terms of precision and recall for the NA12878/HG001 individual using 40 × coverage compared to competitors at a lower cost [67, 71]. The high sequencing accuracy will likely enable the assessment of mosaic variant and cell-free DNA.

A very recent technology called Ultima [72] is capable of producing billions of high-quality sequencing reads (Q30 > 85%) with a length of around ~ 300 bp. The sequencing takes less than 20 h at a very low cost. Such a read dataset was used to call the SNVs and indels (length < 10) of GIAB samples HG001-7 showing an accuracy of 99.6 and 96.4% [72]. The main features of the device that make this possible are open fluidics, optic systems, and their new technology called “mostly natural sequencing chemistry”. This enables bypassing the high cost of sequencing dominated by consumables flow cells and the sequencing reagents. Given the reduced cost, this technology will likely enable large RNA-Seq quantitative analysis.

As aforementioned technologies have recently been presented, no independent studies have yet been performed comparing these to well-established state-of-the-art technologies with each other. Nevertheless, in one study provided by PacBio’s website that was not peer-reviewed, the F1 score (a combination of precision and recall values) of SNVs calling are reported for Element (SNVs:99.5%, indels: 99.6%), Ultima (SNVs:99.6%, indels: 99.6%), Illumina’s NovaSeq (SNVs: 99.7%, indels: 98.1%), and SBB (SNVs: 99.7%, indels: 99.2%), which may not be representative enough [73]. It is noteworthy that assessments of SVs and SNVs calling of different sequencing technologies are affected by benchmark datasets and the included regions across the genome (e.g., tandem repeats and telomers) [11, 13, 14]. Besides, the development of variant calling tools such as Clair [74], Deepvariant [75], Longshot [76], and Sniffles [77] depends on benchmark datasets to measure the performance of new tools. Having comprehensive benchmark datasets including a diverse range of variations indeed provides a more accurate assessment, showing the importance of developing and establishing new datasets.

## Impact of reference genome

One major challenge for creating genomic benchmark datasets is that they are often dependent on the quality of the underlying reference genome. The first human reference genome was released in 2000 covering only the euchromatin fraction of the genome [78, 79]. The human reference genome GRCh38 (a.k.a. hg38) was released by the Genome Reference Consortium in 2013 as a replacement for GRCh37 (a.k.a. hg19) presented in 2009 [80]. GRCh38 was lastly updated in 2022 with a minor new patch, GRCh38.p14. This reference genome includes sequences originally derived from a few individuals with African and European ancestries [80].

The reference genome is a fundamental resource for biomedical research, human genetics, and clinical studies. Interestingly, 151 Mbp of GRCh38 are unknown sequences which are distributed all over the chromosomes [81]. Another shortcoming of GRCh38 is the short arm of chromosome 21 which is represented falsely duplicated and poorly assembled [82]. The impact of such shortcomings is investigated in a study for variant calling performance specifically for CMRG genes, including KCNE1, CBS, CRYAA, TRAPPC10, DNMT3L, and KMT2C [83]. Notably, some initiatives are making efforts to improve the quality of the reference genome, including the T2T consortium [81].

The T2T consortium presents a complete sequence of a human genome, called T2T-CHM13 adding around 200 Mbp to the GRCh38 reference [81]. These cover around 99 protein-coding genes in addition to more than 2000 candidate genes. Besides, T2T corrects several structural errors in the current reference sequence [83, 84]. T2T-CHM13 comprises 3.055 billion bp, 4.5% more than GRCh38. The number of annotated genes has increased from 60,090 to 63,494. In a study by Ji et al. [85], copy number variation (CNV) signal was detected using long-read sequencing data from 41 human individuals across 19 populations in 179 CMRG genes on GRCh38. This number increases to 263 genes on T2T-CHM13. This clearly shows the importance of the reference genome to correctly identify and compare variants.

Despite such progress, some argue that the linear reference genome cannot represent the diverse genetic information of all human populations and discuss that thousands of genetic variations are absent from the reference genome. This leads to the emergence of building human reference pangenomes [43, 86, 87].

Pangenomes could be represented as a genome graph which includes variations among the population. There are several approaches to building such a structure, the most prominent one is based on using a reference genome (as a FASTA file) together with genetic variation (stored in a VCF file) as the alternative paths in the graph [88]. Sequencing reads can be aligned into this reference graph [89], which improves the read alignments [80, 90]. Its advantages were shown previously for characterizing repetitive regions and resolving complex structural variants of medically relevant genes [87]. However, building such graph reference genome and downstream analysis is still computationally expensive, hampering wide applicability in diverse scenarios.

Recently, the Human Pangenome Reference Consortium (HPRC) released the first draft of the human pangenome reference, which encompasses 47 phased diploid assemblies. In comparison to GRCh38, the pangenome contains over 119 Mbp of euchromatic sequences, with approximately 90 Mbp residing in SVs. The quality of the assemblies was evaluated using the benchmark dataset of GIAB v.4.2.1 [91].

The released pangenome represents a significant advancement in the field of genomics. In a recent study, the all-versus-all comparison of the human pangenome (HPRCy1) was beneficial for investigating the short arms of the human acrocentric chromosomes 13, 14, 15, 21, and 22 (SAACs), as these chromosomes share large homologous regions. Specifically, the study demonstrated that SAACs contigs formed a cohesive community characterized by high nucleotide identity [92]. Another study leveraged the efforts of HPRC to systematically study the differences in SNVs between unique and duplicated regions of the human genome, utilizing phased genome assemblies from 47 individuals. The findings indicated a 60% increase in SNVs within segmental duplications compared to unique regions. Notably, more than 23% of these differences were attributed to interlocus gene conversion [93].

Emerging improved reference genomes offer a great opportunity to have more accurate benchmark datasets of genetic variations. However, every new reference brings new challenges with itself. As each benchmark is developed for a specific reference genome and lifting to another reference needs a new manual curation, which is not always straightforward, limiting the applicability of these new datasets in the long run.

## Challenges of benchmark curation: overview of tools

Creating a benchmark dataset is a challenging task. It requires high-quality DNA samples, on which multi-platform sequencing machines should be run. Then, several state-of-the-art software packages should be used to align the read to the reference genome or assemble the genome de novo, call the variant, and analyze the result. For some challenging regions, manual curation is also needed. In this section, we provide an overview of current literature on these steps.

In a typical pipeline for developing benchmark datasets, the first step is to map the DNA sequencing reads to the reference genome. A plethora of tools have been developed for mapping reads to reference including, but not restricted to minimap2 [94], NGMLR [95], BWA-MEM2 [96], LRA [97], Vulcan [98] and Winnowmap2 [99]. After mapping the reads to the reference genome, the next step is calling variants. For calling SNVs and indels, several tools are available such as FreeBayes [100], Strelka [101] and GATK [102] for short-reads DeepVariant [75], PEPPER-Margin-DeepVarian [103] and Clair2 [104] for long reads. While for SVs, Manta [105], DELLY [106] and Parliament2 [107] are used for short reads and Sniffles2 [77], pbsv [108] and CuteSV [109] are available for calling SVs using long reads [17].

Such a pipeline for developing benchmark datasets is tolerant to low coverage, indels, and resolving heterozygosity; thus, more suitable for population-scale studies. However, de novo genome assembly is the most accurate way to give a sample genome representation, but it is computationally demanding [110, 111]. Comparing the assembly to the reference reveals the genomic variations [112]. Some methods use both ONT and HiFi [113] or only ONT [114, 115], while others use HiFi reads solely [42] and still manage to provide a diploid assembly encompassing both haplotypes [33, 113, 116]. Then, one can use Dipcall to identify SNVs based on haplotype-resolved assembly. Comparing assembly methods and technologies for detecting variants is out of the review scope. For more on variant identification and the utility of assembly and alignment approaches, we suggest the reader the review by Mahmoud et al. [17].

In CMRG, Dipcall was used to call the variants from the assembled genome of the sample HG002. CMRG analysis takes advantage of HiFi reads and simultaneously uses fully homozygous human cell lines, resulting in reducing the bias introduced by methods and algorithms towards resolving only simple regions and ignoring challenging regions. Accordingly, both alignment-based variant calling methods and Dipcall were used to produce GIAB v.4.2.1.

Regardless of the method used for identifying SNVs or SVs, there are several tools to benchmark the results. That is done by taking the SNVs or SVs calls and comparing it to the benchmark call set, overcoming minor differences in representation that can happen between different variant calling tools. Thus, these tools help in the assessments of pipelines and medical research, ultimately comparing sequencing methods and centers, and developing new tools.

There are mainly two SNVs benchmarking tools used today. Hap.py [117] is a collection of tools provided by Illumina which is based on the htslib programming library to compare variants. Another package is RTG vcfeval [118], which performs sophisticated comparison of SNVs. The comparison is done at the haplotype level by considering possible genomic sequences when variants are applied to the reference genome. This is not a trivial computational problem, as there are a huge number of combinatorial possibilities for such insertions. They came up with the idea of using dynamic programming, resulting in a practical software for benchmarking the variant set against the truth set.

There are two different techniques for benchmarking: the alignment and the genome assembly approaches. Some of the most well-known tools for the alignment approach include tools like SURVIVOR [119] and Truvari [120]. SURVIVOR performs different tasks like simulation reads and converting different formats to VCF. It also merges and compares SVs within samples and among populations. Finally, Truvari is more advanced, working on a population level, and giving more flexibility in benchmarking. It uses different comparison metrics: SV type, reference distance, sequence and size similarity, reciprocal overlap, and genotype matching. Furthermore, it supports specifying matching stringency of SV size, SV sequence, and reference distance.

One well-known method of genome assembly approach for SVs benchmarking is TT-Mars [121], which uses a haplotype-resolved assembly to assess structural variants. TT-Mars compares call sets to genome assembly and detects how well they represent the assembly, instead of comparing them directly to variant calls. Likewise, the way TT-Mars works makes it less dependent on alignment. Hence, it minimizes the bias from alignment gap parameters originated from alignment tools. To emphasize the importance of the development of benchmarking tools, these are critical to define standard performance metrics and make the process labor free. When it comes to complex structural variants, the challenge is that one SV can be represented in different ways, making the comparison difficult given the wide spectrum of their types and sizes.

## Discussion

In this review, we highlighted the emergence and development of genomics benchmark datasets, while describing the ones currently available in both healthy and patient samples. These datasets provide key insights on the performance of sequencing technologies, as well as analytical methods. We also discussed new alternative references for the human genome that will impact the utility of the so far provided benchmarks. Furthermore, we briefly reviewed new technologies that might lead to further improvements of the currently available benchmark datasets. Altogether, the field of genomic benchmarks is very active with multiple groups (FDA and NIST) introducing ideas and new benchmark sets for variant calling and improving the field of genomics and genetics further [16, 85, 122–124]. This review brings a special focus on the recently released challenging medically relevant genes (CMRGs) benchmark study, which encompasses 386 genes that are challenging due to the complexity of their genomic location. The existence of the CMRGs benchmark dataset explicit the fact that we need to improve state-of-the-art variant comparison methods such as benchmark tools. Benchmark tools give clearer evidence whether variants identified by the tool being tested are present or not in the benchmark dataset, and on the correctness of the variant representation. These are key issues and often not easy to resolve, given the repetitiveness of certain genomic regions that lead variant calling tools to have different ways to represent the genomic variations. The differences in representation can be as easy to identify as insertions vs. duplications, or as difficult as multiple insertions being characterized as one large insertion present at once at a tandem repeat [95].

Despite these direct challenges, there is a huge gain from keeping benchmark datasets up to date and continuing to challenge the genomics and bioinformatics field. With this in mind, there are multiple future opportunities for genomic benchmarks in general. The most obvious one is creating a dataset that combines SNVs, indels, and SVs. While this was first done at the CMRGs benchmark, we are still currently lacking methods to simultaneously identify SNVs and SVs, and thus also benchmark tools that can do the same. One exception might be Dipcall [11], which can provide SNV and SV calls simultaneously. Unfortunately, the vast majority of variant calling methods are focusing on either variant class, and thus our benchmark tools are also specific to each variant type. In terms of variants, benchmark datasets available almost exclusively focus on variants found in germline cells. We reported only one benchmark dataset describing variants identified in a somatic cell lineage as a benchmark set. Despite these issues, we do believe that this gap in benchmark datasets will be closing, hopefully soon. The challenges to do so are multifold, as one not only requires to characterize one sample, but two samples, e.g., of different tissues. Other such benchmark datasets will be needed to obtain key information into the limitations of current available variant detection pipelines and approaches. Together with somatic variants, the interpretation of low variant allele frequency in a single tissue is also an exciting endeavor to pursue for future benchmark datasets. Here, the challenge is to obtain deep coverage datasets and carefully distinguish false signals (PCR or sequencing errors) from true mosaicism. This will be very important not just for cancer, but potentially also for other adult diseases (e.g., brain related) [125]. Besides these challenges, there are of course also the other dimension of characterizing the full human reference genome. Current benchmark datasets are providing a curated variant call set up only to ~ 90% of the human genome (Table 1). Regions such as telomeres, large tandem repeats, and centromeres are often excluded [16, 123]. Furthermore, the T2T-CHM13 reference genome provides further unique regions such as the Acro centromeric regions and small chromosome arms [81, 84]. Due to the utilization of diverse sequencing technologies, algorithms, and software, GIAB stands out as the most comprehensive benchmark dataset among those discussed in this review. As a result, when it comes to developing new variant callers or software for benchmark assessment purposes, we strongly recommend leveraging GIAB as a reference.

Over this review, we focused only on genome-based variant benchmark datasets, but this is of course only the tip of the iceberg. As a different approach, current projects are being designed to address the need for benchmarks designed for mRNA expression. This has multiple challenges, such as the stability of the sample and the impact on the expression rate of genes/isoforms. This will require new benchmark tools to compare the benchmark set of isoforms to that which will be identifiable by other pipelines. Further standards need to be defined in terms of if a missing isoform counts as a false negative or not, which truly depends on the depth of sequencing. This is in contrast to genome approaches where higher coverage is important but will not play such a crucial role as in RNA sequencing [1]. Other benchmark datasets could be thought over, such as methylated DNA or genomic 3D structure, which present many challenges and thus are beyond the scope of this review. Nevertheless, there is a clear need for such datasets to ensure accurate assessment of novel bioinformatics tools at all levels, which likely will lead to an improved and more impactful variant prediction.

With all these innovations in mind, one point that is often discussed is whether to go wide or deep. Meaning, if benchmarks need to be provided across multiple individuals per, e.g., ethnicity at some point (i.e., going wide) or focus on a few individuals (i.e. going deep). Providing a benchmark data set across many individuals would be most comprehensive, as different ethnicities could have different challenges to identify their common alleles. Still, the argument can be made that going deep into a few selected samples is more helpful as it allows benchmarks on the same sample, but across different tissues or essays (DNA, RNA, etc.). Furthermore, a key point is that this would also allow the development of samples carrying pathogenic variants, which are potentially key to pushing the medical genomics field forward. The selection of the individual or disease is of course challenging, and as discussed before, a tumor/normal sample would be probably a good start in this direction. Another aspect is of course also the development of genomic benchmarks across key non-human model species such as plants, animals, and fungi. These could be key for genomics and clinical research, but also for economic reasons.

It is clear that benchmark datasets are key elements of our innovations across genomics and genetics, with a large impact across the human genome-based research. This review provides a snapshot in time on what has been done so far, and we speculate about future endeavors that will push the field forward.

### Supplementary Information

**Additional file 1.** Review History.
