# Supplementary material for: Genomic variant benchmark: if you cannot measure it, you cannot improve it
Source: Genome Biol. 2023 Oct 5;24:221. doi: 10.1186/s13059-023-03061-1 (PMC10552390; doi:10.1186/s13059-023-03061-1)
Supplement: Supplementary file 1 — Additional file 1. Review History. [file 13059_2023_3061_MOESM1_ESM.docx]

Review History

**First round of review**

**Reviewer 1**

**Comments to author:**

In this review, the authors described the importance of benchmark datasets in genomic research. They focused on the several recent benchmark datasets, including the Genome in a Bottle (GIAB) dataset and the challenging medically relevant genes (CMRGs). After describing the current datasets, the authors also reviewed the upcoming changes in the sequencing technologies, new developments in the human reference genome, and benchmarking tools. Overall, this is a well-organized review. The writing is clear and the summary of the benchmark datasets are valuable to the field. I have a few comments/suggestions:

* Ideally a benchmark dataset should reflect the "ground truth" of the genomic variation, with 100% accuracy and sensitivity. However, because of the current technology limitations it is not always the case. Given the review focuses on the benchmark datasets, I believe it is valuable to discuss the definition of "benchmark dataset", as what requirements entail a "benchmark dataset".

Minor comments:

* For the Challenging Medically Relevant Genes (CMRG) set, the number 395 and 386 were used in several places throughout the manuscript. Please clarify the difference.

* Line 178: "they ended up with 395 genes that have at least 90% of the gene body included in GRCh37 or GRCh38." Based on Fig 3B, the statement should be "…395 genes that have <= 90% of the gene body included in GRCh37 or GRCh38."

* Fig 2C-E is presented in the section starting line 213, consider moving the panels to a separate figure in this section.

* Line 249: "Figure 4E" should be "Figure 2E", if the authors decided to keep the Fig 2C-E in the current form.

**Reviewer 2**

**Comments to author:**

General comments

The manuscript by Majidian and colleagues is a review on genomic benchmark datasets. The authors describe datasets that are publicly available including Genome in a Bottle benchmark, focusing on 386 challenging medically relevant genes. While the review is well written and includes the relevant references, the topic is rather narrow and is of interest to a very small group of researchers. Benchmarks are extremely helpful and the review would be of interest to a wider audience if it broader. For example if it includes also additional benchmarks like RNA-seq (see for example Teng et al, Genome Biology 2016, 17:74) or increasingly popular single-cell sequencing data. Alternatively, including statistics with respect to performance in terms of specificity and sensitivity could be a way to go. Currently it is just a list of datasets without recommendations.

Specific comments

1. The chapter on emerging sequencing technologies is nicely written, albeit somehow out of scope since there are no comparative studies. Perhaps if the authors include some applications for which these technologies are useful, it could be helpful for the readers. Alternatively, one could consider omitting the chapter.

2. The chapter on reference genomes has to be updated given the papers recently published (see Nature issue from May10th 2023). Specifically, the human reference pangenomes need a thorough discussion since the reference 82 is published.

**Authors Response**

**Point-by-point responses to the reviewers’ comments:**

Reviewer #1:

In this review, the authors described the importance of benchmark datasets in genomic research. They focused on the several recent benchmark datasets, including the Genome in a Bottle (GIAB) dataset and the challenging medically relevant genes (CMRGs). After describing the current datasets, the authors also reviewed the upcoming changes in the sequencing technologies, new developments in the human reference genome, and benchmarking tools. Overall, this is a well-organized review. The writing is clear and the summary of the benchmark datasets are valuable to the field. I have a few comments/suggestions:

*Response: We appreciate the reviewer for providing valuable feedback and insightful suggestions on our paper. Furthermore, the reviewer's suggestions have enhanced the quality and depth of our paper.*

* Ideally a benchmark dataset should reflect the "ground truth" of the genomic variation, with 100% accuracy and sensitivity. However, because of the current technology limitations it is not always the case. Given the review focuses on the benchmark datasets, I believe it is valuable to discuss the definition of "benchmark dataset", as what requirements entail a "benchmark dataset".

*Response: We would like to thank the reviewer for the insightful comment, We have thoroughly addressed this issue and incorporated the suggested addition into our work, we added the following paragraph in the Discussion:*

*“A genome variant benchmark dataset serves as a vital resource for evaluating the effectiveness and accuracy of newly developed variant callers, whether they focus on small or large genomic alterations. This dataset comprises known curated genomic variants. To ensure a comprehensive assessment, it is crucial for the benchmark dataset to include relevant information about the specific genomic regions associated with these variants. This distinction is essential as it helps differentiate these regions from those that were not considered in the benchmark dataset, such as regions that are not assembled or lack support from multiple callers. This information is typically captured and represented in a BED file format, ensuring that researchers have precise details regarding the genomic coordinates and regions associated with each variant.”*

*And this section:*

*“Although attaining 100% accuracy and sensitivity is challenging due to technological limitations, benchmark datasets strives to provide the highest accurate representation for genomic variations. To achieve this, creating a benchmark dataset involves utilizing diverse sequencing technologies such as long-reads, short-reads, and linked-reads, each with varying insert sizes and high coverage. Additionally, a range of variant calling tools and methods that rely on both mapping and assembly techniques are integrated to mitigate methodological biases. The key ingredients, however, is the manual and often wet lab assessment of the variance across a community effort [14].*

*In the construction of a benchmark dataset, several other criteria are carefully considered. For instance, regions where callers exhibit systematic errors or misrepresent genotypes are typically excluded [11]. Similarly, areas demonstrating high complexity may also be excluded to ensure a more accurate representation of genomic variations [14]. These selection criteria help to enhance the quality and reliability of the benchmark dataset, providing researchers with a robust and comprehensive resource for evaluating variant calling algorithms.”*

Minor comments:

* For the Challenging Medically Relevant Genes (CMRG) set, the number 395 and 386 were used in several places throughout the manuscript. Please clarify the difference.

* Line 178: "they ended up with 395 genes that have at least 90% of the gene body included in GRCh37 or GRCh38." Based on Fig 3B, the statement should be "…395 genes that have <= 90% of the gene body included in GRCh37 or GRCh38."

*Response: We thank the reviewer for pointing this out. Indeed it's 395 medically relevant genes if we sum up the genes from Grch37+38. It's 386 medically relevant genes on Grch38 alone. We made this now clearer in the manuscript. The sentence now reads:*

*“Only 5,027 (4,697 autosomal genes) have coordinates on GRCh38 genome assembly, and they ended up with 395 genes that have at maximum 90% of the gene body included in either GRCh37 or GRCh38 (386 if evaluating GRCh38 alone).”*

* Fig 2C-E is presented in the section starting line 213, consider moving the panels to a separate figure in this section.

* Line 249: "Figure 4E" should be "Figure 2E", if the authors decided to keep the Fig 2C-E in the current form.

*Response: We have divided Figure 2 into two separate figures and made corresponding updates to the main text. As a result, Figure 2 now focuses specifically on comparing benchmarks in terms of the number of citations and reference inclusion. On the other hand, Figure 4 is dedicated to describing the Challenging Medically Relevant Genes (CMRG) list.*

Reviewer #2:

The manuscript by Majidian and colleagues is a review on genomic benchmark datasets. The authors describe datasets that are publicly available including Genome in a Bottle benchmark, focusing on 386 challenging medically relevant genes. While the review is well written and includes the relevant references, the topic is rather narrow and is of interest to a very small group of researchers. Benchmarks are extremely helpful and the review would be of interest to a wider audience if it broader.

*Response: Thank you for your valuable feedback on our manuscript. We appreciate your positive comments about the quality of the writing and the inclusion of relevant references.*

For example if it includes also additional benchmarks like RNA-seq (see for example Teng et al, Genome Biology 2016, 17:74) or increasingly popular single-cell sequencing data.

*Response: We have taken careful consideration of your suggestions, and we have now incorporated the available RNA-seq benchmarks into our review.*

Alternatively, including statistics with respect to performance in terms of specificity and sensitivity could be a way to go. Currently it is just a list of datasets without recommendations.

*Response: We acknowledge the importance of providing performance statistics, such as specificity and sensitivity, for the benchmark datasets which would be possible for simulated datasets or when side information is available. We added a paragraph regarding this important matter.*

*Besides, in order to expand the evaluation of benchmarks quantitatively, we depicted a new figure as a comparison of available benchmarking datasets in terms of variants they cover (SNV, indels, SV) in Figure 2C (Provided below).*

*Finally, we added a paragraph in the Discussion section providing recommendations on the available benchmarks.*

*“Due to the utilization of diverse sequencing technologies, algorithms, and software, GIAB stands out as the most comprehensive benchmark dataset among those discussed in this review. As a result, when it comes to developing new variant callers or software for benchmark assessment purposes, we strongly recommend leveraging GIAB as a reference.”*

Specific comments

1. The chapter on emerging sequencing technologies is nicely written, albeit somehow out of scope since there are no comparative studies. Perhaps if the authors include some applications for which these technologies are useful, it could be helpful for the readers. Alternatively, one could consider omitting the chapter.

*Response: We appreciate the reviewer's valuable feedback and have taken it into consideration. In response to this feedback, we have made additions to the "emerging sequencing technologies" section of the manuscript. These additional paragraphs now emphasize the pivotal role of these new technologies in driving advancements within the field.*

2. The chapter on reference genomes has to be updated given the papers recently published (see Nature issue from May10th 2023). Specifically, the human reference pangenomes need a thorough discussion since the reference 82 is published.

*Response: We have extended the section on the human pangenome and described the newly published papers. We added the following section to the manuscript:*

*“Recently, the Human Pangenome Reference Consortium (HPRC) released the first draft of the human pangenome reference, which encompasses 47 phased diploid assemblies. In comparison to GRCh38, the pangenome contains over 119 Mbp of euchromatic sequences, with approximately 90 Mbp residing in SVs. The quality of the assemblies was evaluated using the benchmark dataset of GIAB v.4.2.1.*

*The released pangenome represents a significant advancement in the field of genomics. In a recent study, the all-versus-all comparison of the human pangenome (HPRCy1) was beneficial for investigating the short arms of the human acrocentric chromosomes 13, 14, 15, 21 and 22 (SAACs), as these chromosomes share large homologous regions. Specifically, the study demonstrated that SAACs contigs formed a cohesive community characterized by high nucleotide identity (Guarracino et al. 2023). Another study leveraged the efforts of HPRC to systematically study the differences in SNVs between unique and duplicated regions of the human genome, utilizing phased genome assemblies from 47 individuals. The findings indicated a 60% increase in SNVs within segmental duplications compared to unique regions. Notably, more than 23% of these differences were attributed to interlocus gene conversion (Vollger et al. 2023).”*

**Second round of review**

**Reviewer 1**

The authors addressed all my previous comments and the manuscript has been improved in multiple aspects. I have no further comments.

**Reviewer 2**

The authors addressed all the issues raised in the previous review and thereby considerably improved the manuscript. Specifically, inclusion of additional benchmarks, performance metrics as well as modifications of the manuscript as suggested were carried out.

**Authors Response**

**Point-by-point responses to the reviewers’ comments:**

*Response: We would like to thank the reviewers for their effort and feedback that was important to improve the manuscript.*
